# Intra‐Abdominal Hemorrhage Following Endoscopic Retrograde Cholangiopancreatography in a Patient with a Cascade Stomach: A Case Report

**DOI:** 10.1002/deo2.70345

**Published:** 2026-05-13

**Authors:** Masashi Miura, Naoki Okano, Yamato Ohara, Ryo Ikegami, Wataru Ujita, Hiroki Nakagawa, Yusuke Kimura, Kensuke Takuma, Takahisa Matsuda, Yoshinori Igarashi

**Affiliations:** ^1^ Department of Internal Medicine Division of Gastroenterology and Hepatology Omori Medical Center Toho University Tokyo Japan

**Keywords:** bleeding, cascade stomach, complication, endoscopic retrograde cholangiopancreatography, intra‐abdominal hemorrhage

## Abstract

Intra‐abdominal hemorrhage is an extremely rare complication of endoscopic retrograde cholangiopancreatography (ERCP). We report a case of intra‐abdominal bleeding that occurred after ERCP for choledocholithiasis with cholangitis in a patient with a cascade stomach. A man in his 70s underwent ERCP for severe acute cholangitis due to a common bile duct stone. The procedure was initially attempted using a TJF‐260 V duodenoscope (Olympus); however, insertion into the duodenum was difficult because of the stomach cascade. The scope was changed to a JF‐260 V (Olympus), and duodenal access was successfully achieved using a large‐diameter sliding tube (ST‐CB1; Olympus), followed by biliary stent placement. No sphincterotomy or other invasive procedure was performed. Two hours after the procedure, the patient developed severe epigastric pain, hypotension, and progressive anemia. Contrast‐enhanced computed tomography (CT) revealed a large hematoma centered on the gastrosplenic ligament, indicating an intra‐abdominal hemorrhage that was likely caused by a traction injury resulting from scope manipulation. No evidence of contrast extravasation was found, and conservative management was performed. The patient's condition improved with blood transfusion and discontinuation of aspirin, and follow‐up CT demonstrated resolution of the hematoma. Inserting a scope can be challenging owing to anatomical deformities such as a cascade stomach, and excessive torque or traction may result in vascular injury. Careful insertion using a sliding tube and positional adjustment are essential, with awareness that intra‐abdominal hemorrhage, although rare, can be a serious complication.

## 1 Introduction

Endoscopic retrograde cholangiopancreatography (ERCP) is widely used to treat pancreatobiliary diseases. The major complications of ERCP include pancreatitis, cholangitis, perforation, and bleeding. The incidence of bleeding has been reported to be approximately 0.3%–2%, and most cases involve intraluminal bleeding associated with endoscopic sphincterotomy or papillary balloon dilation [1]. In contrast, extraluminal bleeding, such as intra‐abdominal hemorrhage, is extremely rare, and only a limited number of cases have been reported. While intraluminal bleeding can often be readily identified by clinical signs, such as melena, intra‐abdominal hemorrhage may present with nonspecific symptoms and can occur in a delayed manner several hours after the procedure, making diagnosis challenging. Mild hemorrhage cases may improve with conservative management; however, severe hemorrhage cases may require the identification of the bleeding source and endovascular intervention, which can potentially be fatal. Here, we report a rare case of intra‐abdominal hemorrhage following ERCP performed for choledocholithiasis‐associated cholangitis in a patient with a cascade stomach.

## 2 Case Report

A man in his 70s was hospitalized for treatment of acute heart failure. His medical history included cerebral infarction, and he was taking aspirin. During hospitalization, he developed fever and right upper quadrant abdominal pain with a positive Murphy's sign. On examination, he was alert with a temperature of 38.6°C, blood pressure of 103/54 mmHg, and a heart rate of 118 beats per minute. Laboratory tests revealed C‐reactive protein 1.8 mg/dL, total bilirubin 4.4 mg/dL, AST 3109 U/L, ALT 829 U/L, ALP 579 U/L, γ‐GTP 899 U/L, amylase 110 U/L, white blood cell count 4.5 × 10^3^/µL, hemoglobin 12.5 g/dL, and platelet count 8.9 × 10^4^/µL.

**FIGURE 1:**
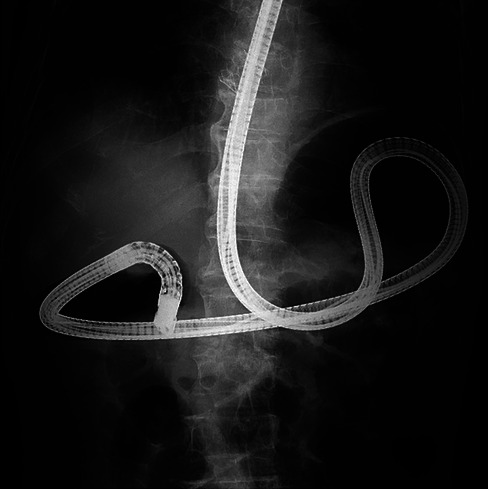
The procedure began with a TJF‐260 V duodenoscope (Olympus); however, due to gastric deformity (cascade stomach), the scope looped in the fundus, making duodenal intubation difficult.

**FIGURE 2:**
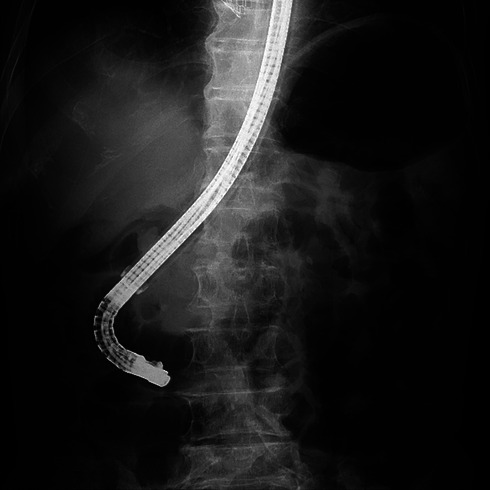
(a) The papilla was successfully reached after changing to a JF‐260 V scope and using a large‐caliber sliding tube (ST‐CB1; Olympus).

**FIGURE 3:**
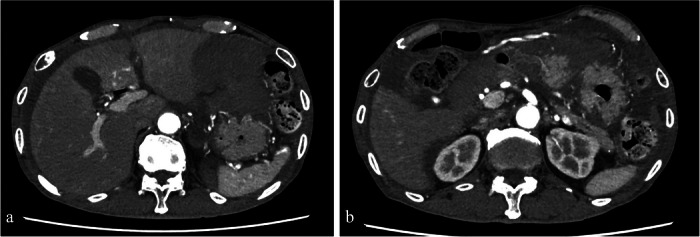
(a, b) Contrast‐enhanced computed tomography (CT) reveals a large hematoma around the gastrosplenic ligament and a localized hypoenhanced area in the posterior gastric wall. No contrast extravasation or vascular abnormality was identified on imaging.

**FIGURE 4:**
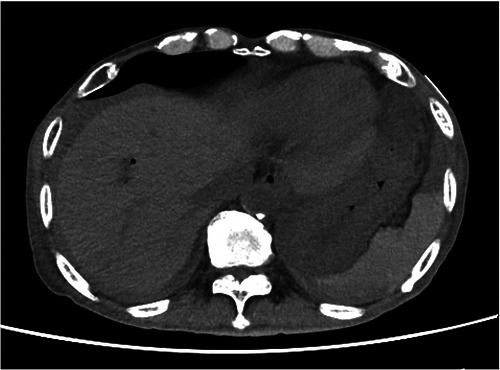
The hematoma around the gastrosplenic ligament showed improvement on follow‐up computed tomography (CT) obtained 2 days after endoscopic retrograde cholangiopancreatography (ERCP).

Computed tomography (CT) revealed a 4‐mm stone in the distal common bile duct, accompanied by dilation of the upstream bile duct. Based on the Tokyo Guidelines 2018, the patient was diagnosed with severe (Grade III) acute cholangitis due to choledocholithiasis, and emergency ERCP was performed. Aspirin therapy had been discontinued prior to ERCP. The procedure was initiated using a side‐viewing duodenoscope (TJF‐260 V; Olympus, Tokyo, Japan). However, duodenal intubation was difficult because the scope was looped in the gastric fundus due to a cascade stomach (Figure 1). After approximately 10 min of unsuccessful attempts, the scope was exchanged for a side‐viewing endoscope (JF‐260 V; Olympus, Tokyo, Japan), and the patient was repositioned to the left lateral position. A large‐diameter sliding tube designed for colonoscopy (ST‐CB1; Olympus, Tokyo, Japan) was then used. This exchange was performed to allow the use of the sliding tube, as the JF‐260 V could be accommodated within it. As a result, access to the papilla was successfully achieved, and a biliary stent was placed (Figure 2). Endoscopic sphincterotomy and stone extraction were deferred due to the patient's poor general condition and antiplatelet therapy. Approximately two hours after ERCP, the patient developed severe epigastric pain and hypotension (54/36 mmHg). Laboratory tests revealed a marked decrease in hemoglobin to 6.8 g/dL. Contrast‐enhanced CT demonstrated an extensive perigastric hematoma centered around the gastrosplenic ligament (Figure 3a,b). The attenuation measured approximately 60–70 HU, and no vascular malformation, aneurysm, or other vascular abnormality was identified on contrast‐enhanced CT. An intra‐abdominal hemorrhage caused by vascular injury due to excessive gastric traction during scope insertion was suspected. The exact bleeding vessel could not be identified because no contrast extravasation was observed, and angiography was not performed. However, the location and distribution of the hematoma suggested injury to the short gastric arteries within the gastrosplenic ligament. Conservative management was initiated with blood transfusion and aspirin discontinuation. The patient required six units of red blood cell transfusions. Hemoglobin levels increased from 6.8 to 10.8 g/dL after transfusion and remained stable thereafter. Follow‐up CT 2 days later showed a reduction in the hematoma (Figure 4). After stabilization, repeat ERCP was successfully performed using the same assisted technique, resulting in complete stone removal.

## 3 Discussion

Bleeding associated with ERCP‐related procedures is most commonly intraluminal and usually related to endoscopic sphincterotomy or papillary balloon dilation. However, intra‐abdominal hemorrhage is an extremely rare extraluminal complication, with only a few reported cases. Intra‐abdominal hemorrhage often develops several hours after the procedure. While mild cases may resolve with conservative treatment, severe cases may require identification of the bleeding point and endovascular therapy, which may lead to fatal outcomes [2]. Kuno et al. reported a case of intra‐abdominal hemorrhage caused by a short gastric artery injury following ERCP that required endovascular treatment, suggesting that excessive traction and torque during scope manipulation could lead to vascular injury [3]. Ishigaki et al. reported a case of left gastric artery bleeding following diagnostic endoscopic ultrasonography, and similarly concluded that traction stress on the gastric wall induced by the endoscope contributed to vascular injury [4]. These reports and the present case share a common mechanism of mechanical vascular injury caused by endoscopic manipulation, as well as a delayed onset occurring several hours after the procedure. However, to the best of our knowledge, no previous report has described intra‐abdominal hemorrhage as a complication of ERCP‐related procedures that is specifically associated with a cascade stomach. In the present case, the gastric axis angulation due to the cascade stomach resulted in excessive stretching of the gastric fundus during duodenoscope insertion, which may have injured the short gastric arteries or adjacent vessels within the gastrosplenic ligament. Anatomically, the short gastric arteries run from the upper stomach toward the splenic hilum and are susceptible to overstretching when the gastric fundus is excessively distended or pulled [5]. The use of antiplatelet therapy with aspirin may also have contributed to hematoma expansion and delayed hemostasis. In patients with a cascade stomach, scope insertion is often difficult because of loop formation and concentration of traction forces at the gastric fundus, necessitating technical modifications. Previous technical reports suggested that gastric decompression by suction, careful torque manipulation, and patient repositioning, such as the left lateral position, may help reduce excessive gastric extension. As an adjunctive technique, Saito et al. demonstrated that the use of a large‐diameter sliding tube designed for colonoscopy stabilized the scope axis and reduced excessive angulation, enabling safe insertion into the descending duodenum even in patients with a cascade stomach [6]. Furthermore, Doba et al. proposed an alternative technique to reinforce scope rigidity by inserting forceps through the working channel when large‐diameter sliding tube use is not feasible, thereby minimizing loop formation associated with a cascade stomach [7]. Aabakken et al. emphasized that in patients with anatomical gastric deformities, including a cascade stomach, selective use of fluoroscopic loop reduction techniques, large‐diameter sliding tube, or scope rigidity‐enhancing methods is crucial for achieving safe and controlled duodenoscope insertion [8]. Taken together, these findings and the present case suggest that adjunctive techniques aimed at stabilizing the scope axis, reinforcing scope rigidity, and minimizing excessive traction are important for reducing the risk of vascular injury and preventing serious complications, such as intra‐abdominal hemorrhage, during ERCP in patients with a cascade stomach.

Here, we report a rare case of ERCP‐related intra‐abdominal hemorrhage caused by excessive scope traction in a patient with a cascade stomach. In patients with anatomical gastric deformities such as a cascade stomach, excessive traction and torque during endoscopic manipulation can lead to vascular injury. Careful scope manipulation and proactive use of adjunctive techniques are essential to minimize gastric traction and prevent serious complications. Although intra‐abdominal hemorrhage is rare, it should be considered in patients who develop abdominal pain or progressive anemia after ERCP.

## Author Contributions

Masashi Miura conceived the study, managed the case, collected and analyzed the data, and drafted the manuscript. All co‐authors provided clinical support and supervision, and reviewed and approved the final manuscript.

## Funding

The authors have nothing to report.

## Ethics Statement

Written informed consent was obtained from the patient for the publication of this case report and the accompanying images. Institutional Review Board approval was not required for this study.

## Conflicts of Interest

The authors declare no conflicts of interest.

## Data Availability

The data that support the findings of this study are available from the corresponding author upon reasonable request.
